# Supervised Contrastive Learning and Intra-Dataset Adversarial Adaptation for Iris Segmentation

**DOI:** 10.3390/e24091276

**Published:** 2022-09-10

**Authors:** Zhiyong Zhou, Yuanning Liu, Xiaodong Zhu, Shuai Liu, Shaoqiang Zhang, Yuanfeng Li

**Affiliations:** 1 College of Computer Science and Technology, Jilin University, Changchun 130012, China; 2 Key Laboratory of Symbolic Computation and Knowledge Engineering of Ministry of Education, Jilin University, Changchun 130012, China; 3 College of Biological and Agricultural Engineering, Jilin University, Changchun 130012, China

**Keywords:** contrastive learning, adversarial adaptation, iris segmentation, deep learning

## Abstract

Precise iris segmentation is a very important part of accurate iris recognition. Traditional iris segmentation methods require complex prior knowledge and pre- and post-processing and have limited accuracy under non-ideal conditions. Deep learning approaches outperform traditional methods. However, the limitation of a small number of labeled datasets degrades their performance drastically because of the difficulty in collecting and labeling irises. Furthermore, previous approaches ignore the large distribution gap within the non-ideal iris dataset due to illumination, motion blur, squinting eyes, etc. To address these issues, we propose a three-stage training strategy. Firstly, supervised contrastive pretraining is proposed to increase intra-class compactness and inter-class separability to obtain a good pixel classifier under a limited amount of data. Secondly, the entire network is fine-tuned using cross-entropy loss. Thirdly, an intra-dataset adversarial adaptation is proposed, which reduces the intra-dataset gap in the non-ideal situation by aligning the distribution of the hard and easy samples at the pixel class level. Our experiments show that our method improved the segmentation performance and achieved the following encouraging results: 0.44%, 1.03%, 0.66%, 0.41%, and 0.37% in the *Nice1* and 96.66%, 98.72%, 93.21%, 94.28%, and 97.41% in the *F1* for UBIRIS.V2, IITD, MICHE-I, CASIA-D, and CASIA-T.

## 1. Introduction

With the increasing importance of information security in the information society, physiological feature recognition technology and behavioral feature recognition technology have emerged. They are used on a large scale in access control, identity recognition, and other scenarios. The main biometric features currently studied in academia and industry are fingerprint, face, voice, and iris [1].

**Figure 1:**
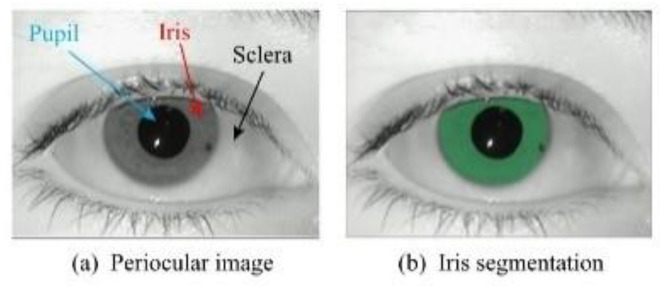
Periocular structure and iris segmentation [3].

The iris is an internal organ of the human body [2], and Figure 1a shows the iris in the eye along with other periocular structures [3]. Fingerprints can extract only a few feature points, faces have a dozen, while irises can extract more than two hundred feature points [4]. Therefore, the iris has been widely used in our daily life as one of the most accurate, trustworthy, and promising biometric technologies [5].

Iris recognition usually consists of five steps: iris acquisition, iris quality evaluation, iris segmentation, iris feature extraction, and iris feature matching. Iris segmentation plays a very important role in iris recognition [6], and the final recognition rate depends on the performance of segmentation [7]. Iris segmentation is divided into generalized segmentation and narrow segmentation. Narrow iris segmentation is used to generate a binary mask to extract the iris pixel region as shown in Figure 1b, where the green region indicates the valid iris pixels. Our approach focuses on the latter, i.e., iris semantic segmentation.

Since the errors in iris segmentation are passed to the subsequent feature extraction and feature matching [8], it is of great significance to develop effective and efficient iris segmentation methods. Currently, the two types of traditional methods that exist are divided into two major categories. One category is the group of boundary-based methods represented by the integral differential method [9] and the Hough transform method [10]. They separate the iris region of interest by locating the boundaries of the pupil, sclera, and eyelid. The other category is the group of pixel-based methods such as extracting Zernike moments to construct SVM to discriminate iris pixels [11] and designing location and color features using ANN for iris pixel classification [12]. However, these traditional methods require complex prior knowledge and extensive pre- and post-processing and are not suitable for non-ideal iris acquisition environments.

With the great success of deep learning techniques represented by convolutional neural networks (CNNs) in computer vision, it has become popular to design end-to-end iris segmentation networks with better segmentation performance than traditional methods. However, deep learning techniques applied to iris semantic segmentation face two challenging problems:

1. Training of deep convolutional neural networks requires a large amount of data, whereas the available dataset of iris images is very limited and is not enough to effectively train the network [7]. In addition, labeling data for the iris semantic segmentation task is expensive and time-consuming as it requires dense pixel-level annotations. The common practice for training with limited annotated data is to first pretrain using commonly used large classical databases such as ImageNet [13] and then fine-tune the network. However, ImageNet is designed for academic research and not for commercial applications. It may be not suitable for developing practical iris recognition products. In addition, ImageNet does not effectively help the semantic segmentation problem of non-natural images [14];
2. Iris acquisition is usually unconstrained and non-cooperative, so the quality of the obtained images is very limited, which can lead to degraded performance of segmentation [15]. For example, the images may contain non-uniform illumination, bokeh, blurring, reflections, and eyelid/eyelash occlusion [16].

Most of the existing deep-learning-based iris segmentation methods [17,18,19,20,21] do not solve the above problems well, and the ideal segmentation performance of [18,19,20] is heavily dependent on large-scale data. These data must be iris pixels accurately labeled by hand, which is time-consuming and expensive. The studies [19,20] use several augmentation technologies including cropping, resizing with interpolation, horizontal flipping, horizontal translation, and vertical translation to expand the data by 12 times. These studies significantly increase the computational complexity and the storage overhead of model training. Study [17] captures local texture details and global structural information from multiple scales but may misclassify for some similar noisy pixels. Study [21] only learns irregular iris shapes using dense blocks, and its segmentation performance is degraded by illumination variations. The studies [17,21] ignore the differences in the feature distribution of the dataset itself. They cannot overcome the degraded performance in the face of the non-ideal iris acquisition condition. To better address the above problems, motivated by self-supervised learning [22,23], we designed a pretraining paradigm for iris semantic segmentation. It increases the separability of inter-class pixels and the compactness of intra-class pixels by modeling the relationship between similar and dissimilar pixels in the feature space in different images. We did not use additional ImageNet and data augmentation to improve the segmentation performance of a limited amount of annotated dataset. According to domain adaptation in transfer learning, a big distribution gap between the source domain data and the target domain data can lead to a decrease in the generalization ability of the model. The performance of the model also decreases when there are also differences in the distribution within the dataset [24]. Reference [25] argues that the source and target domain data have different distributions due to the domain gap, which in turn leads to different output entropy values for the same model in different domains. The entropy minimization objectives push the model’s decision boundaries toward the low-density regions of the target domain distribution in the prediction space. For some iris datasets acquired in unconstrained environments, the distribution of iris images within the dataset is inconsistent. Motivated by entropy minimization, we used entropy to encode the output space of the model to define the level of difficulty of samples with different distributions. We assumed that the model produces a high entropy value for the segmentation output of hard samples that contain more noise, i.e., the output of the model is of low confidence. The model produces a low entropy value for the segmentation output of clean easy samples, i.e., the output of the model is of high confidence. Therefore, we used the entropy-based intra-dataset adversarial adaptation at the level of pixel class to reduce the gap between low- and high-quality iris feature distributions within the database to improve the segmentation performance in unconstrained environments.

In summary, our main contributions are summarized as:

(1) We propose a three-stage iris segmentation training algorithm. It offers an alternative training pipeline for iris segmentation networks on small and non-ideal datasets;
(2) The supervised contrastive learning is proposed to pretrain the iris segmentation feature extraction model to bring features of similar pixels close to each other and to keep the features of dissimilar pixels away from each other. It reduces the need for large amounts of dense pixel-level labeled data and additional large-scale data such as ImageNet;
(3) Intra-dataset adversarial adaptation is proposed to align the distribution of sample features with different noise levels, improving the robustness of the model on non-ideal datasets;
(4) Our approach achieves state-of-the-art results in some metrics such as F1 score, Nice-1, mIoU, etc. on several commonly used datasets including those with few samples;
(5) To the best of our knowledge, this work pioneers the use of contrastive learning and domain adaptation to improve iris segmentation performance.

## 2. Related Work

With the growing demand for identification and access control, the rapid development of iris recognition technology has been promoted. As one of the key parts, iris segmentation exists in two major categories of methods, including traditional image processing methods and deep-learning-based data-driven methods. These methods are summarized in Table 1. Traditional image processing methods can be subdivided into two categories: boundary-based methods and pixel-based methods. 

The boundary-based approaches locate the inner and outer iris boundaries based on the presence of gradient changes in the borders of the iris and pupil, iris, and sclera, and the geometry of the borders. John Daugman uses the integro-differential operator to integrate the gradient in the circumference along the radial direction, and the parameters corresponding to the maximum value of the integration are used as the iris boundary parameters [9]. In the system of Wildes et al., the Hough transform is used to segment the iris region by binarizing the edge points and using the edge points to vote on the boundary parameters. In addition, the parameters with the most votes are determined as the parameters of the iris boundary [10]. Radman et al. use both the integro-differential operator and Hough transform to segment the iris region in the visible light environment [26]. The Hough transform has been further improved in [27,28]. Study [27] reduces the parametric search space of the Hough transform without reducing the accuracy and accelerates the detection of boundaries using the one-dimensional space of the radius. Uhl A et al. apply an adaptive Hough transform to find the center of the most discriminative concentric circle using both gradient direction and gradient magnitude [28].

Unlike the boundary-based approaches, the pixel-based approaches classify iris pixels and non-iris pixels by a binary classifier based on images with rich pixel features such as texture, color, and location. Well-known pixel-based methods rely mainly on low-level-intensity pixel features to discriminate iris pixels from other pixels by graph cut based on entropy minimization [29]. Banerjee et al. represent the image as a Markov random domain and obtain the localization results using ellipse fitting based on the geometry of a modified graph cut version of garbcut [30]. Radman et al. use HOG-SVM to obtain some columns of labeled pixels, and the segmentation results of the iris are obtained by cellular automata which evolved through GrowCut [31].

It is worth noting that boundary-based approaches rely on the contrast at regional transitions and thus localize borders using gradients and contours. However, pixel-based approaches focus on low-level visual features to build binary classifiers. Some methods combine the advantages of both approaches to obtain better segmentation results. Tan et al. use an eight-neighbor connection-based clustering method to roughly label iris pixels and non-iris pixels. In addition, a novel integrodifferential constellation is used to precisely locate the inner and outer iris boundaries [32]. On the contrary, Kumar et al. use the random walker method to coarsely localize the boundaries and graph-based modeling to accurately distinguish iris regions [33].

However, current traditional image processing methods require a series of complex pre-processing and post-processing operations, which are not conducive to developing practical iris recognition products. Manual features relying on a priori knowledge lack generalizability in the face of different acquisition environments. In addition, segmentation errors generated when faced with unconstrained scenes are passed to subsequent steps, leading to a dramatic decrease in iris recognition rate.

In recent years, artificial intelligence and deep learning have made great progress in image processing tasks due to the strong increase in GPU computing power. In addition, more and more deep convolutional neural networks have achieved SOTA on large datasets [37,38,39,40]. The neural network architecture search for designing effective and efficient networks for semantic segmentation has also attracted the attention of researchers [41]. In addition to applications in natural images, deep learning is beneficial for building powerful diagnostic and predictive systems using CT scan images such as early prediction of lung cancers [42]. Transformers as alternative architectures for CNNs can effectively address any image segmentation task (panoptic, instance, or semantic) [43]. They have made progress in biomedical image segmentation [44]. Study [45] presents a comprehensive review of the important loss functions for biomedical image segmentation. Deep learning algorithms have also been widely applied to iris segmentation, which far outperform traditional image processing algorithms in terms of segmentation performance. Liu et al. [17] propose hierarchical convolutional neural networks (HCNNS) and multiscale fully convolutional neural networks (MFCNS) for end-to-end optimization without pre- and post-processing. MFCNS fuse shallow local features and deep global features to capture coarse and fine details and are more robust in the face of noise than HCNNS. Bazrafkan et al. [18] merge four different full convolutional networks by SPDNN at the layer level using graph theory calculation and graph contraction to obtain a U-net-like network without pooling facing low-quality iris images. Wang et al. [15] propose an elaborate partitioned network with attention modules to obtain masked and parameterized inner and outer boundaries by optimizing a unified multitasking network. The double attention densely connected network (DADCNET) proposed by Chen et al. [34] contains two attention modules and improves skip connections that replace the corresponding GT images using mask images segmented by deep learning methods. Wang et al. [35] propose a lightweight fully connected neural network, using a weighted loss, multi-level-feature dense-fusion module, with multi-supervised training of a multi-scale image, and a generative adversarial network to improve mobile iris segmentation performance. Miron et al. [36] propose a U-net convolutional neural network that contains model downscaling to improve efficiency.

Most of the above deep learning approaches work on designing a complex dedicated network for iris segmentation to solve the non-cooperative environment problem and designing a complex dedicated network for iris segmentation increases the difficulty of iris recognition system development. Some expand a large amount of training data and use the additional large-scale dataset to avoid model overfitting, which increases the storage and computational pressure. Most of them don’t consider the distribution gap within the unconstrained dataset. Adversarial adaptation can effectively align the domain distribution [46]. Study [47] achieves global cross-domain alignment and generates reliable pseudo-labels for the target domain. Study [48] learns the domain-invariant feature for visual location via adversarial adaptation. Our proposed approach achieves good performance on a small amount of data without data expansion on existing network models using only a simpler contrastive training strategy that is generalizable. Secondly, the feature distribution gap of different noise-level samples within the dataset is reduced by an adversarial adaptation framework, which is robust in the face of unconstrained environments.

## 3. Technical Details

### 3.1. Overview of the Proposed Method

**Figure 2:**
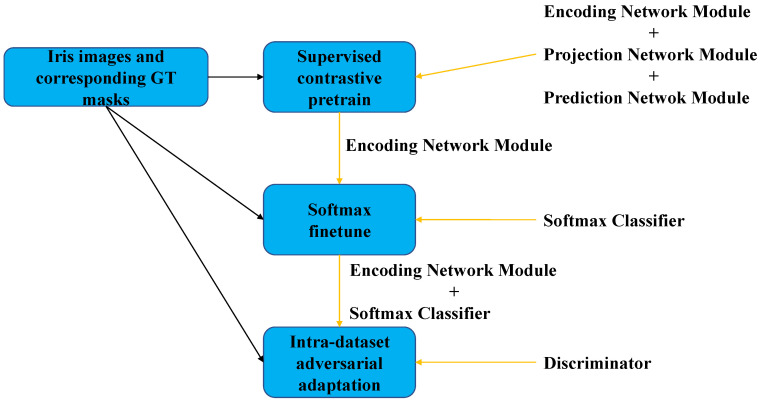
Overview of the proposed method.

The complete flowchart of our iris segmentation algorithm training is shown in Figure 2, which is divided into three stages: supervised contrastive pretraining of the iris segmentation network, finetuning of the iris segmentation network, and intra-dataset adaptation of the iris segmentation network. Instead of using a pixel-wise cross-entropy loss, a pixel-wise contrastive loss is used to pretrain the iris segmentation network in the first stage. The model consists of the feature extraction part of the existing segmentation network as an encoding module, a feature extractor followed by a projection module, and a prediction module followed by a projection module. To better learn the representation of the image, a channel attention mechanism is added between the encoding module and the projection module, and between the projection module and the prediction module [49]. In addition, the purpose of this stage is to make intra-class pixels compact and inter-class pixels separable with a limited amount of labeled data. The second stage adds a pixel-wise softmax classifier to the encoder obtained in the first stage and uses a cross-entropy loss to fine-tune the whole network. The third stage uses an intra-dataset adversarial adaptation framework, where the entire network in the second stage acts as a generator to obtain the full image as input and outputs a semantic segmentation mask map. In addition, a discriminator is used to predict the labels of the difficult and easy samples. This phase trains the generator and discriminator iteratively to align the feature distributions of iris images of different quality. It reduces the internal gap of the dataset, and further improves the performance of the segmentation model.

### 3.2. Supervised Contrastive Learning for Iris Segmentation Pretraining

**Figure 3:**
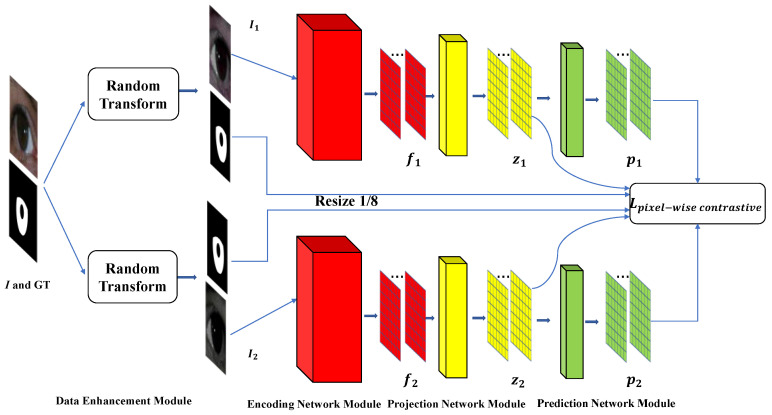
Supervised contrastive learning framework.

The purpose of recent self-supervised learning is to obtain the representation of images, and the representation is obtained by deep-convolutional-neural-network output. It mainly uses the auxiliary task (pretext) to mine the self-supervised information from large-scale unsupervised data so that the network can learn valuable representations for downstream tasks [50,51,52]. Specifically, they use an enhanced version of instances to form positive pairs and other randomly sampled instances to form negative pairs to compensate for contrastive loss [51]. Simsiam [53] is a relatively simple framework that does not require negative sample pairs, large batches, and momentum encoders [23]. In addition, it outperforms the optimal self-supervised learning and supervised learning algorithms on ImageNet image classification tasks at a lower epoch iteration of training. We extend the existing image level-based contrastive self-supervised learning algorithm to the pixel-based level, which is more suitable for the dense prediction of image semantic segmentation. The entire model framework is shown in Figure 3. The framework for supervised contrastive learning consists mainly of the following:

(1) Data enhancement module: Given a batch of iris images and segmentation GTs, a series of spatial geometric transformations, color jitter, and drop and blurring operations are used to process the batch data as Random Transform. The iris images are first randomly cropped with the segmentation mask in the ratio [0.5,2.0] relative to the size of the original image and are then resized to 513 × 513 using a bilinear interpolation algorithm. The iris images and GTs are also randomly flipped left and right at the same time. Color jitter is to randomly adjust the brightness, contrast, saturation, and hue of the image. The color drop is a random graying of the image. The random blur operation uses a mean filter, gaussian blur, median blur, bilateral filter, and box blur.

(2) Encoding network module: Unlike previous self-supervised learning architectures [22,23,53] that use ResNet as the backbone, we directly use DeepLabV3 [37] based on ResNet50 [54] without the last convolutional layer as the backbone to ensure highly stable pixel mapping feature representation. The output feature space resolution of the encoding network module is 1/8 of the input, and the output feature channel, i.e., the dimension of the feature representation per pixel, is 128.

(3) Projection network module: We use three 1 × 1 convolution layers with BN and 128 channels. Each convolutional layer uses Leaky-ReLu, except for the third convolutional layer.

(4) Prediction network module: The module consists of two 1 × 1 convolution layers with BN, the first with 32 output channels and the second with 128 output channels.

(5) Attention module: The channel attention mechanism [49] is inserted between the encoding network module and the projection network module, and between the projection network module and the prediction network module. The importance of different channel features can be automatically learned between the modules.

(6) Supervised pixel-wise contrastive loss: To extend the self-supervised learning image classification to dense prediction tasks at the pixel level, the data samples in our contrastive loss calculation are the pixels in the image. Given the input sample image *I*, two views $I_{1}$ and $I_{2}$ of the sample image I are obtained by the data enhancement module. Next, the encoding network module takes two views as the input to extract feature maps $f_{1}$ and $f_{2}$, respectively. Continually, feature maps $f_{1}$ and $f_{2}$ are fed to the attention module and the projection module to obtain feature maps $z_{1}$ and $z_{2}$. It continues to obtain feature maps $p_{1}$ and $p_{2}$ through the attention module and the prediction network module. The cosine similarity function is used to measure the similarity between pixel feature vectors and is formulated as:

(1) $$H\left(p_{1}^{i},z_{2}^{j}\right)=\frac{p_{1}^{i}}{∥p_{1}^{i}{∥}_{2}}\cdot \frac{z_{2}^{j}}{∥z_{2}^{j}{∥}_{2}}$$

where $p_{1}^{i}$ denotes the feature vector on $p_{1}$ corresponding to the original image I at pixel i, and $z_{2}^{j}$ denotes the feature vector on $z_{2}$ corresponding to I at pixel j. To avoid the problem of pattern collapse, we borrowed stop-gradient, an important component of Simsiam [53]. It is formulated as follows:

(2) $$H\left(p_{1}^{i},stopgrad\left(z_{2}^{j}\right)\right)$$

where stopgrad indicates that $I_{2}$ cannot receive the gradient from $z_{2}$. Our supervised learning pixel-wise contrastive loss is formulated as:

(3) $$L_{pixel-wise\;contrastive}=-\frac{1}{N^{I_{1}}}\overset{N^{I_{1}}}{\underset{i}{\sum }}\frac{1}{N_{y_{i}^{I_{1}}}^{I_{2}}}\overset{N^{I_{2}}}{\underset{j=1}{\sum }}1\left[y_{i}^{I_{1}}=y_{j}^{I_{2}}\right]\log\left\{\frac{exp\left(H\left(p_{1}^{i},stopgrad\left(z_{2}^{j}\right)\right)\right)}{\sum_{k=1}^{N^{I_{2}}}H\left(p_{1}^{i},stopgrad\left(z_{2}^{k}\right)\right)}\right\}\;-\frac{1}{N^{I_{2}}}\overset{N^{I_{2}}}{\underset{i}{\sum }}\frac{1}{N_{y_{i}^{I_{2}}}^{I_{1}}}\overset{N^{I_{1}}}{\underset{j=1}{\sum }}1\left[y_{i}^{I_{1}}=y_{j}^{I_{2}}\right]\log\left\{\frac{exp\left(H\left(p_{2}^{i},stopgrad\left(z_{1}^{j}\right)\right)\right)}{\sum_{k=1}^{N^{I_{1}}}H\left(p_{2}^{i}stopgrad\left(z_{1}^{k}\right)\right)}\right\}$$

where $N^{I_{1}}$ denotes the total number of pixels of $I_{1}$, $y_{i}^{I_{1}}$ denotes the label of the ith pixel of $I_{1}$, $N_{y_{i}^{I_{1}}}^{I_{2}}$ denotes the number of labels of the ith pixel of $I_{1}$ in $I_{2}$, and $1\left[y_{i}^{I_{1}}=y_{j}^{I_{2}}\right]$ denotes that when the label of the ith pixel of $I_{1}$ is equal to the label of the jth pixel of $I_{2}$, it takes 1, otherwise, it takes 0.

The training strategy of contrastive learning is as follows: the framework is given a batch of full images and corresponding GTs of the raw training set as the input. In addition, the output of the prediction network module and the corresponding GTs after the data augmentation module are used to compute $L_{pixel-wise\;contrastive}$ to train the whole framework. After the contrastive learning is completed, we only use the encoding network module as the iris semantic segmentation feature extractor. A convolution layer is added as the pixel-wise classifier to construct the overall iris segmentation network. It takes the full images and corresponding GTs of size 513 × 513 as the input on the original training set, and we fine-tune the overall iris segmentation network using pixel-wise cross-entropy loss.

### 3.3. Intra-Dataset Adaptation for Iris Segmentation

#### 3.3.1. Global Spatial Level Adaptation

To solve the problem of degraded iris segmentation performance due to the large gap in feature distribution caused by noise factors such as uneven illumination, spectral reflection, eyelid occlusion, eye hair interference, and off-axis within the dataset, we draw on adversarial domain adaptation [1]. Study [1] argues that the source and target domain data have different distributions due to the domain gap, which in turn leads to different output entropy values for the same model in different domains. Since the hard and easy sample data share a strong similarity in semantic layout, we define the feature distribution of the hard and easy samples indirectly through the entropy value of the model structure space output. An adversarial training approach is used to make the entropy distribution of the noisy samples within the dataset similar to that of the clean samples, while keeping the entropy value of the segmentation feature map of the low-noise samples at a low level. The approach reduces the distribution gap within the dataset, forcing the dataset to approximately satisfy the independent identical distribution and improving the performance of the segmentation.

**Figure 4:**
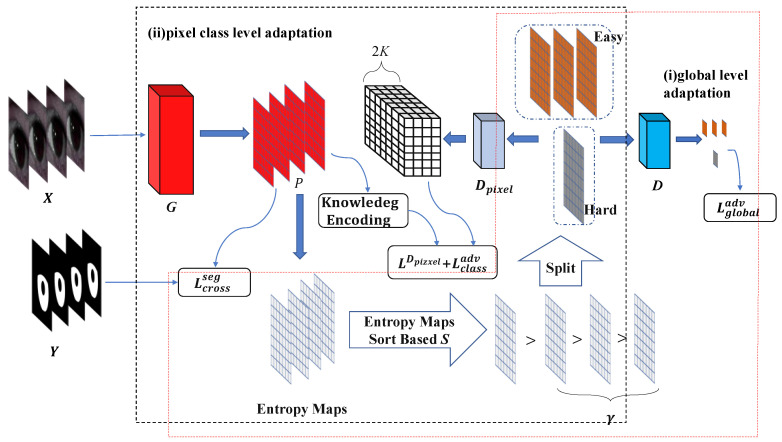
Two intra-dataset adaptation frameworks.

As shown in Figure 4i, the framework is given an iris sample $X\in R^{H\times W\times 3}$ with a pixel label map $Y\in {\left\{0,1\right\}}^{H\times W}$. $Y^{\left(h,w\right)}$ denotes a label of a pixel (h,w) as a one-hot vector. The iris sample X is input to the segmentation model G to generate a soft segmentation feature map P=softmax(G(X)), which is the predicted probability of the pixel category. Given X and the corresponding Y, the segmentation network G is optimized using pixel-wise cross-entropy loss as follows:

(4) $$L_{cross}^{seg}\left(X\right)=-\overset{H}{\underset{h}{\sum }}\overset{W}{\underset{w}{\sum }}\overset{K}{\underset{c}{\sum }}Y^{\left(h,w,c\right)}\cdot \log\left(P^{\left(h,w,c\right)}\right)$$

where K = 2 indicates that the number of categories for pixel classification is 2, i.e., iris regions and non-iris regions.

The quality of the images of the iris samples in the dataset is difficult to annotate manually. To quantify the difficulty of the iris samples, we draw on Shannon’s information entropy principle [55]. Therefore, the entropy value of the soft segmentation output of the segmentation model at the pixel (h,w) is defined as follows:

(5) $$E^{\left(h,w\right)}\left(X\right)=-\overset{K}{\underset{c}{\sum }}-P^{\left(h,w,c\right)}\cdot \log\left(P^{\left(h,w,c\right)}\right)$$

To define the relationship between the value of the output entropy map and the distribution of easy and hard samples, we rank the confidence levels of the iris samples using the following formula:

(6) $$S=\sqrt[{h\cdot w}]{\overset{h}{\underset{i=1}{\prod }}\overset{w}{\underset{j=1}{\prod }}E^{\left(i,j\right)}}$$

We set a ratio coefficient $\gamma =\frac{\left|X_{easy}\right|}{\left|X\right|}$ for dividing the hard and easy samples, where |X| denotes the total number of samples in the entire iris training dataset, and $\left|X_{easy}\right|$ denotes the number of easy samples. Using γ, we then divide the iris training dataset based on the S sort into easy and hard samples.

To align the distribution shift of features of the easy and difficult samples within the dataset, we train a binary discriminator D to predict the domain labels of the easy and difficult samples in the dataset. We set the domain labels of the easy samples to 0 and the domain labels of the difficult samples to 1. The loss function of the discriminator is formulated as:

(7) $$L_{global}^{adv}\left(X_{easy},X_{hard}\right)=\log\left(1-D\left(E_{hard}\right)\right)+\log\left(D\left(E_{easy}\right)\right)$$

where $E_{hard}=\underset{h,w}{\sum }E^{\left(h,w\right)}\left(X_{hard}\right)$ denotes the entropy map of difficult samples and $D\left(E_{hard}\right)$ denotes the sigmoid output obtained by using the entropy map $E_{hard}$ as the input to the discriminator.

The entire adversarial adaptation learning process at the global level is as follows: The iris segmentation network is used as G in adversarial adaptive learning. We firstly freeze G, $\underset{D}{\min}L_{global}^{adv}$ to train D. Second, we freeze D, $\underset{G}{\min}L_{cross}^{seg}+\underset{G}{\max}L_{global}^{adv}$ to train G. Finally, we repeat the above steps until the model converges. The learning process is summarized in Algorithm 1.

**Algorithm 1**: Global spatial level adaptation**Input**: iris data X, pixel label Y,  segmentation network G, training epochs T, ratio coefficient γInitialize: binary discriminator D
For t = 1,…,T do  Unfreeze the *D* and freeze the *G*  Compute the logit maps: P=softmax(G(X))
  $\mathrm{Compute}\;\mathrm{the}\;\mathrm{entropy}:\;E^{\left(h,w\right)}\left(X\right)=-\overset{K}{\underset{c}{\sum }}-P^{\left(h,w,c\right)}\cdot \log\left(P^{\left(h,w,c\right)}\right)$
  Sort the logit maps P based the $S=\sqrt[{h\cdot w}]{\overset{h}{\underset{i=1}{\prod }}\overset{w}{\underset{j=1}{\prod }}E^{\left(i,j\right)}}$
  Split the iris data X corresponding to sorted P into $X_{hard}$ and $X_{easy}$ by ratio coefficient γ
  Compute the *D*’s loss: $L_{global}^{adv}\left(X_{easy},X_{hard}\right)=\log\left(1-D\left(E_{hard}\right)\right)+\log\left(D\left(E_{easy}\right)\right)$
  $\underset{D}{\min}L_{global}^{adv}$ to train D
  Unfreeze the *G* and freeze the *D*  $\mathrm{Compute}\;\mathrm{the}\;\mathrm{cross}-\mathrm{entropy}:\;L_{cross}^{seg}\left(X\right)=-\overset{H}{\underset{h}{\sum }}\overset{W}{\underset{w}{\sum }}\overset{K}{\underset{c}{\sum }}Y^{\left(h,w,c\right)}\cdot \log\left(P^{\left(h,w,c\right)}\right)$
  $\underset{G}{\min}L_{cross}^{seg}+\underset{G}{\max}L_{global}^{adv}$ to train G


#### 3.3.2. Pixel Class Level Adaptation

The entropy distribution of difficult samples is indirectly minimized by making the entropy distribution of difficult samples similar to that of easy samples. The feature distribution of the samples in the global feature space is aligned, but the feature distribution in the pixel class space is not considered. Previous work [56] argues that aligning only the edge feature distribution, i.e., the global feature space distribution, does not guarantee a significant reduction in the expected error in the target domain. Study [57] also points out that the global feature distribution of the two domains is aligned, but some samples are still misclassified. To solve the above problem, we exploited the class information of iris and non-iris regions in the adversarial training framework. Hence the feature distribution shift at the pixel class level is aligned. In the adversarial training process, the discriminator models the complex structural information at the pixel class level in addition to distinguishing between difficult and easy samples to obtain the class-level alignment.

As shown in Figure 4ii, we extend the domain label output of discriminator D from a one-dimensional vector to an output of the same size as the discriminator input with 2K channels, and the extended discriminator is $D_{pixel}$.

The class constraint knowledge is extracted from the output logits of the segmentation network. It is used as a supervisory signal to enable the global distribution of features of the hard and easy samples to be aligned while considering fine-grained alignments at the class level. The class constraint knowledge can be formulated as:

(8) $$\mu_{i}=\frac{e^{a_{i}/T}}{\sum_{j=1}^{K}e^{a_{j}/T}}$$

where $a_{i}$ denotes the ith channel map of logits output from the segmentation network and T is a hyperparameter making the class constrain the salience of knowledge.

After adding class-level adaptation to global-level adaptation, the loss function of optimizing the discriminator is formulated as:

(9) $$L^{D_{pixel}}\left(X_{easy},X_{hard}\right)=-\overset{K}{\underset{i=1}{\sum }}\mu_{i}^{\left(easy\right)}\log P_{D_{pixel}}^{\left(i\right)}\left(E_{easy}\right)+\overset{K}{\underset{j=1}{\sum }}\mu_{j}^{\left(hard\right)}\log P_{D_{pixel}}^{\left(j+K\right)}\left(E_{hard}\right)$$

where $P_{D_{pixel}}^{\left(i\right)}$ denotes the ith channel of the soft output of the discriminator $D_{pixel}$ and $\mu_{i}^{\left(easy\right)}$ denotes the ith channel of the category-constrained knowledge of easy samples.

By fooling the discriminator with the output of the segmentation network, class-level adversarial loss allows the segmentation network to learn distribution-invariant features of the hard and easy samples at the global level and class level. The class-level adversarial loss is formulated as:

(10) $$L_{class}^{adv}\left(X_{easy},X_{hard}\right)=-\overset{K}{\underset{j=1}{\sum }}\mu_{j}^{\left(hard\right)}\log P_{D_{pixel}}^{\left(j+K\right)}\left(E_{hard}\right)$$

The whole adversarial adaptation learning process at the pixel class level is that the iris segmentation network as G, $\underset{D_{pixel}}{\min}L^{D_{pixel}}$ and $\underset{G}{\min}L_{cross}^{seg}+L_{class}^{adv}$ iteratively optimize $D_{pixel}$ and G. The learning process is summarized in Algorithm 2.

**Algorithm** 2: Pixel class level adaptation**Input**: iris data X, pixel label Y, segmentation network G, training epochs φ, ratio coefficient γInitialize: extended discriminator $D_{pixel}$
For t = 1,…, φ do  Unfreeze the $D_{pixel}$ and freeze the G
  
Compute the logit maps: P=softmax(G(X))
  Extract class constraint knowledge: $\mu_{i}=\frac{e^{a_{i}/T}}{\sum_{j=1}^{K}e^{a_{j}/T}}$
  Compute the $D_{pixel}$’s loss: $L^{D_{pixel}}\left(X_{easy},X_{hard}\right)=-\overset{K}{\underset{i=1}{\sum }}\mu_{i}^{\left(easy\right)}\log P_{D_{pixel}}^{\left(i\right)}\left(E_{easy}\right)+\overset{K}{\underset{j=1}{\sum }}\mu_{j}^{\left(hard\right)}\log P_{D_{pixel}}^{\left(j+K\right)}\left(E_{hard}\right)$
  $\underset{D_{pixel}}{\min}L^{D_{pixel}}$ to train $D_{pixel}$
  Unfreeze the G and freeze the $D_{pixel}$
  
$\mathrm{Compute}\;\mathrm{the}\;\mathrm{cross}-\mathrm{entropy}:\;L_{cross}^{seg}\left(X\right)=-\overset{H}{\underset{h}{\sum }}\overset{W}{\underset{w}{\sum }}\overset{K}{\underset{c}{\sum }}Y^{\left(h,w,c\right)}\cdot \log\left(P^{\left(h,w,c\right)}\right)$
  Compute the class level adversarial loss: $L_{class}^{adv}\left(X_{easy},X_{hard}\right)=-\overset{K}{\underset{j=1}{\sum }}\mu_{j}^{\left(hard\right)}\log P_{D_{pixel}}^{\left(j+K\right)}\left(E_{hard}\right)$
  $\underset{G}{\min}L_{cross}^{seg}+L_{class}^{adv}$ to train G


## 4. Experiments

### 4.1. Datasets

**Figure 5:**
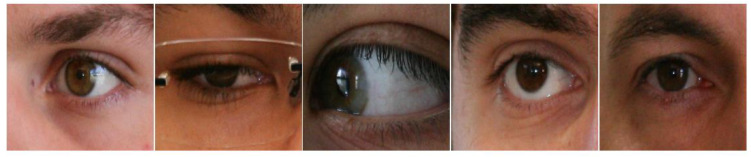
Some iris samples of UBIRIS.v2 dataset.

**Figure 6:**
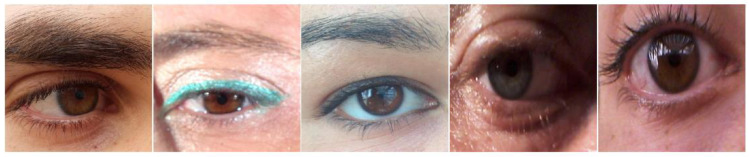
Some iris samples of MICHE-I dataset.

**Figure 7:**
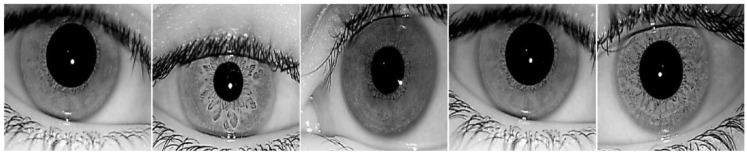
Some iris samples of IITD dataset.

**Figure 8:**
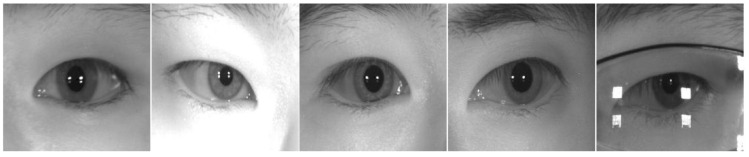
Some iris samples of CASIA-Iris-Distance dataset.

**Figure 9:**
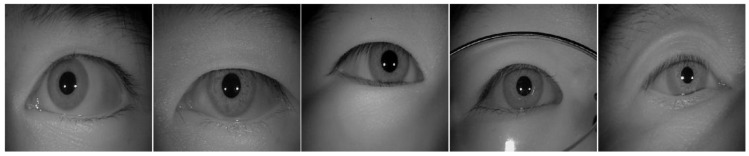
Some iris samples of CASIA-Iris-Thousand dataset.

To validate our proposed method more fairly and effectively, we used two visible light datasets, MICHE-I [58] and UBIRIS.v2 [59], and three near-infrared light datasets, IITD [60], CASIA-Iris-Distance [61], and CASIA-Iris-Thousand [61], which are commonly used in the literature. Figure 5, Figure 6, Figure 7, Figure 8 and Figure 9 show some sample images. These datasets except CASIA-Iris-Thousand were all acquired in unconstrained environments, and CASIA-Iris-Thousand dataset contains rich noise such as illumination variation and spectacle occlusion. In addition, all the datasets are small, which are very suitable for verifying the effectiveness of our proposed algorithm. We followed a consistent data division protocol in the biometrics community.

UBIRIS.v2, containing 11,102 iris samples from 261 subjects, has a low aggressive acquisition increasing the heterogeneity of the dataset, e.g., iris occlusion, reflection, declination, motion blur rotation, etc. We took 2250 images from 50 subjects and randomly selected 1575 images as the training set and the remaining 675 images as the test set.

MICHE-I, a multiracial dataset, was acquired by iris-owning selfies using a mobile device under uncontrolled conditions, in which 1262 images were acquired using an iPhone 5, 1297 images were acquired by a Samsung Galaxy S4, and 632 images were acquired by a Samsung Galaxy Tab2. Similar to UBIRISV2, there were multiple noises. We randomly selected 680 images as the training set and 191 images as the test set.

IITD, which contains 2240 iris samples from 224 subjects, has dense eyelash occlusion noise in the iris region because the collected race is Indian. We randomly selected 1568 images as the training set and 672 images as the test set.

CASIA-D, an abbreviation of CASIA-Iris-Distance, contains 2576 iris samples from 142 subjects, collected at a distance of more than 3 m. In addition, the subjects had moving behaviors during the collection, as well as glasses obscuration. We randomly selected 296 images as the training set and 99 images as the test set.

CASIA-T, an abbreviation of CASIA-Iris-Thousand, contains 20,000 iris samples from 1000 subjects whose images contain noise such as glasses, spectral changes, and so on. We randomly selected 14,000 images as the training set and 6000 images as the test set.

### 4.2. Implementation Details

#### 4.2.1. Evaluation Metrics

We used four metrics, *MIOU*, *F1*, pixel accuracy (*PA*), and *Nice1*, commonly used in the literature on iris segmentation, to evaluate the performance of the segmentation algorithm.

For *MIOU*, we need to calculate *IOU* for each category of pixels first. *IOU* calculates the ratio of the intersection and the concatenation between the set of the predicted number of pixel categories and the set of the number of pixel categories of the real label. For a particular category of pixels, the *IOU* of pixels in that category is calculated as follows:

(11) $$IOU_{k}=\frac{N_{TP}}{N_{TP}+N_{FP}+N_{FN}}$$

where the class of *IOU* for which the class k is calculated is labeled as a positive class and the other classes are labeled as a negative class, $N_{TP}$ denotes the number of pixels that are correctly predicted as a positive class, $N_{FP}$ denotes the number of pixels that are incorrectly predicted as a positive class, and $N_{FN}$ denotes the number of pixels incorrectly predicted as a negative class.

After we calculate the *IOU* of each category, we take the average value to calculate *MIOU*. The range of this value is [0,1]. The larger the value is, the higher the performance of segmentation is. The formula of *MIOU* is as follows:

(12) $$MIOU=\frac{1}{K}\overset{K-1}{\underset{k=0}{\sum }}IOU_{k}$$

where *K* is the total number of categories that represent pixels.

The *F*1 score measures the change in pixel prediction of false positives and false negatives, and the range of this value is [0,1]. A larger *F*1 indicates a smaller percentage of incorrect pixel predictions and a better performance of segmentation. It can be calculated from precision and recall. The precision measures the purity of positive predictions about the ground truth, and the recall measures the completeness of positive predictions about the ground truth. *Precision*, *recall*, and *F*1 are calculated as follows:

(13) $$precision=\frac{N_{TP}}{N_{TP}+N_{FP}}$$

(14) $$recall=\frac{N_{TP}}{N_{TP}+N_{FN}}$$

(15) $$F1=\frac{2\cdot precision\cdot recall}{precision+recall}$$

The *Nice*-1 metric, which is an evaluation protocol of the NICE-I Contest [62], measures the average segmentation error rate by calculating the pixel-wise logical heterogeneous operations of Ground Truth and predicted Mask. In addition, the smaller the value is, the better the segmentation performance is. The *Nice*-1 metric is calculated as follows:

(16) $$Nice1=\frac{1}{T\cdot M\cdot N}\overset{T}{\underset{t=0}{\sum }}\overset{M}{\underset{m=0}{\sum }}\overset{N}{\underset{n=0}{\sum }}G_{t}\left(m,n\right)⊗M_{t}\left(m,n\right)$$

where T denotes the number of images, M denotes the height of the image, *N* denotes the width of the image, $G_{t}\left(m,n\right)$ denotes the Ground Truth of the pixel value of the mth column and nth row of the tth image, $M_{t}\left(m,n\right)$ denotes the predicted Mask of the pixel of the mth column and nth row of the tth image, and ⊗ denotes the logical XOR operation, i.e., 0 for equal pixel values, 1 for unequal ones.

#### 4.2.2. Model Structure

The segmentation network *G* used for our adaptation at the global spatial level and class level is DeeplabV3 based on ResNet50 with a softmax classifier. The discriminator *D* at the global spatial level consists of 6 convolutional layers with channel numbers {16,32,64,128,256,1}, 4 × 4 kernels, stride of 2, and padding of 1. Each convolution layer is followed by a Leaky-ReLU operation parameterized by 0.2 except for the last layer. The discriminator $D_{pixel}$ at the pixel class level consists of four convolutional layers. Firstly, the entropy map is first passed through two convolutional layers with channel numbers {64,32}, 3 × 3 kernels, stride of 1 and padding of 1, and the output is then input to two convolutional layers with channel numbers of {2,2}, 3 × 3 kernels, stride of 1, and padding of 1 in parallel, and finally the two output feature maps are concatenated in the direction of the channel to obtain the output with 4 channels.

#### 4.2.3. Training Setup

The training stage is performed on a training set of each of the five datasets. When performing supervised contrastive learning, we use an Adam optimizer with batch size of 32, a base learning rate of $5\times 10^{-5}$, beta1 of 0.9, and beta2 of 0.999. CosineAnnealingDecay is used to decay the learning rate. The network does not load a pretrained model but is trained from scratch. After supervised contrastive learning, pixel-wise cross-entropy is used to finetune the network with an SGD optimizer with batch size of 16, a fixed learning rate of 0.0001, momentum of 0.9, and weight decay of $10^{-5}$. When performing global spatial level adaption, we train the discriminator *D* using an Adam optimizer with a fixed learning rate of $10^{-4}$, beta1 of 0.9, and beta2 of 0.999, and we train the segmentation network *G* using an SGD optimizer with a fixed learning rate of $2\times 10^{-4}$, momentum of 0.9, and weight decay of $10^{-4}$. When adapting at the pixel class level, we train the discriminator $D_{pixel}$ using an Adam optimizer with a base learning rate of $10^{-4}$, beta1 of 0.9, and beta2 of 0.999, and we train the segmentation network *G* using an SGD optimizer with a base learning rate of $2\times 10^{-4}$, momentum of 0.9, and weight decay of $10^{-4}$. The discriminator $D_{pixel}$ and segmentation network *G* both are trained using the Poly learning rate decay strategy, and the difficulty sample scaling factor is set to γ = 0.6. More importantly, iris semantic segmentation has a remarkable imbalance between the number of iris pixels and the number of non-iris pixels, which is prone to pixel prediction bias [18]. Therefore, we use modified pixel-wise cross-entropy with category frequency weights, where the category frequency is calculated by computing the median of the pixel frequencies, called Media Frequence Balancing [63]. In addition, the category frequency weights for each dataset are shown in Table 2. To make the experimental results more statistically significant, we performed five training sessions for each experiment, and the test results of all evaluation indicators are the mean values of the five training sessions.

### 4.3. Ablation Experiments

To verify that our proposed supervised contrastive learning and intra-dataset adaptation training methods are effective in improving the performance of iris segmentation, we designed three sets of experiments: supervised contrastive learning vs. ImageNet Pretraining, global spatial level adaptation vs. pixel class level adaptation, and the effect of the combination of different strategies on segmentation performance.

#### 4.3.1. Impact of Supervised Contrastive Learning on Segmentation Performance

From Scratch indicates training with cross-entropy loss as the target on the iris segmentation dataset without using any pretrained model. ImageNet+FineTune indicates that the ResNet50-based backbone of the segmentation network uses the pretrained model from ImageNet and then the entire network is fine-tuned with cross-entropy loss. SCL+FineTune indicates that the segmentation network is first pretrained using supervised contrastive learning, and then fine-tuning of the entire network is performed on the iris segmentation training set with cross-entropy loss. The cross-entropy training process for the third method is the same as that for the first two methods. The evaluation results obtained by inference of the trained model on the test set are shown in Table 3. For all dataset scenarios, supervised contrastive pretraining significantly improved on every evaluation metric compared to learning from scratch without pretraining. Compared to ImageNet pretraining, supervised contrastive pretraining was higher on IITD, CASIA-D, MICHE-I, and CASIA-T for all metrics. In addition, in the UBIRIS.v2 dataset, *F1* and *MIOU* were higher except for *PA*, which was slightly lower by 0.02, but *F1* could more comprehensively evaluate the performance of the segmentation algorithm. From the *F1* metric, compared with learning from scratch without pretraining, SCL+FineTune had the largest improvement of 7.27% on dataset CASIA-D and a decent improvement of only 0.91% on dataset CASIA-T. The CASIA-T training set had a much larger number of samples than CASIA-D. Therefore, it can be intuitively shown that our supervised contrastive pretraining algorithm could significantly alleviate the overfitting problem of the model in the case of a limited number of the dataset. These results clearly show the effectiveness of our supervised contrastive pretraining algorithm.

#### 4.3.2. The Impact of Intra-Dataset Adaptation on Segmentation Performance

GLA denotes global spatial level adaptation, and From Scratch+GLA denotes the global spatial level adaptation is performed after learning with cross-entropy loss by no ImageNet pretraining first. CLA denotes pixel class level adaptation, and From Scratch+GLA denotes the pixel class level adaption is performed after learning with cross-entropy loss by no ImageNet pretraining first. As shown in Table 4, on all data sets, both adaptation algorithms improved their values on each evaluation metric compared to From Scratch. In addition, the pixel class-level adaption outperformed the global spatial level adaption in all metrics on all datasets. Because these datasets all have a lot of noise, there was a distribution gap between samples. Once the feature distributions of the samples with more noise and less noise were registered, the dataset was forced to approximately satisfy the independent and identical distribution. Thus, the intra-dataset adaptation improved the segmentation performance.

#### 4.3.3. Comparison of Performance under Different Strategies

The effect of the combination of different strategies on the segmentation performance is shown in Table 5. The three ✗ represents that the segmentation model was trained from scratch without loading the pretrained model. For all datasets, all metrics of the segmentation performance were maximized when we used the combined SCL + GLA strategy. This was because the combined strategy not only increased the compactness of intra-class pixels and separability of inter-class pixels, but also aligned the feature distribution at the class pixel level among samples of different noise degrees. Therefore, the segmentation performance for iris images was greatly improved.

### 4.4. Qualitative Result and Analysis

**Figure 10:**
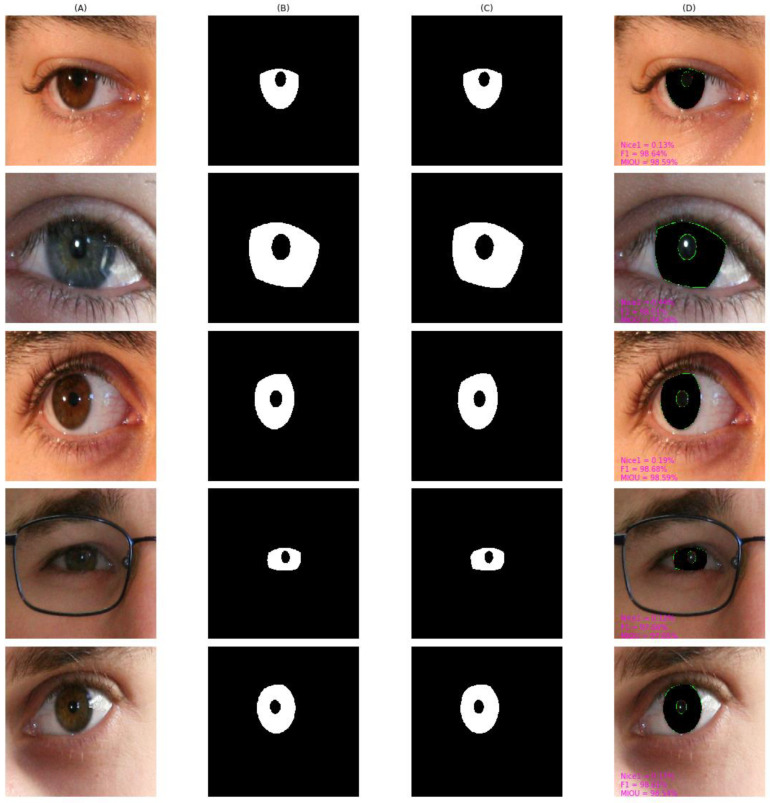
The segmentation result from UBIRIS.v2 achieved by our proposed method. (**A**) original image, (**B**) Groundtruth mask, (**C**) segmentation mask, (**D**) segmentation result, whose green pixels, red pixels, and black pixels represent false positive pixels, false negative pixels, and true positive pixels, respectively.

**Figure 11:**
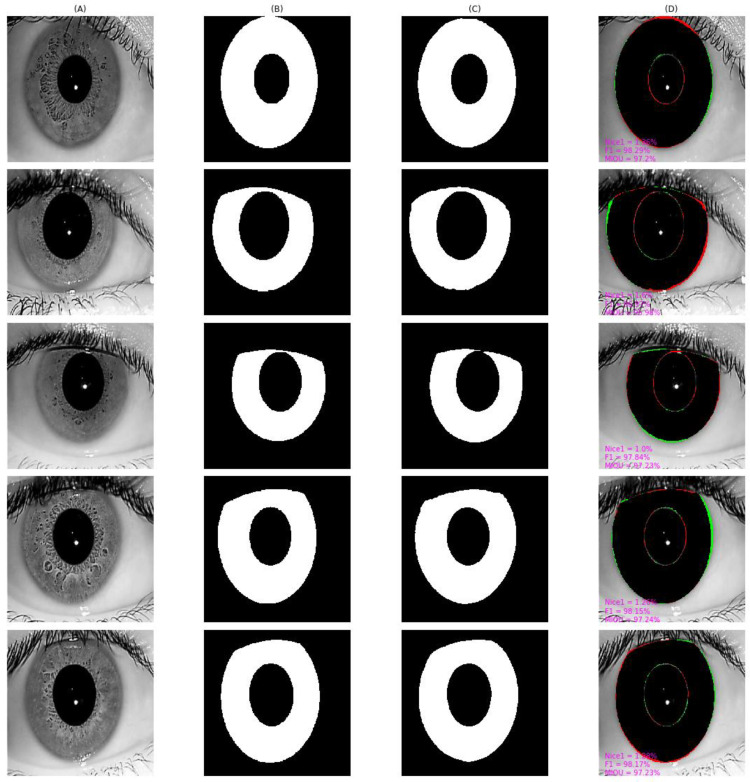
The segmentation result from IITD achieved by our proposed method. (**A**) original image, (**B**) Groundtruth mask, (**C**) segmentation mask, (**D**) segmentation result, whose green pixels, red pixels, and black pixels represent false positive pixels, false negative pixels, and true positive pixels, respectively.

**Figure 12:**
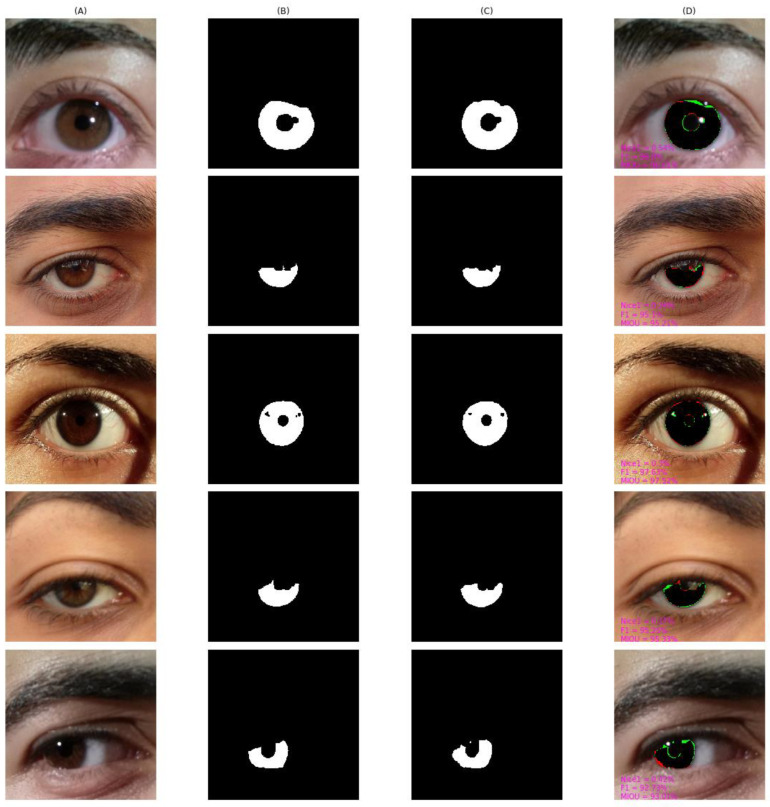
The segmentation result from MICHE-I achieved by our proposed method. (**A**) original image, (**B**) Groundtruth mask, (**C**) segmentation mask, (**D**) segmentation result, whose green pixels, red pixels, and black pixels represent false positive pixels, false negative pixels, and true positive pixels, respectively.

**Figure 13:**
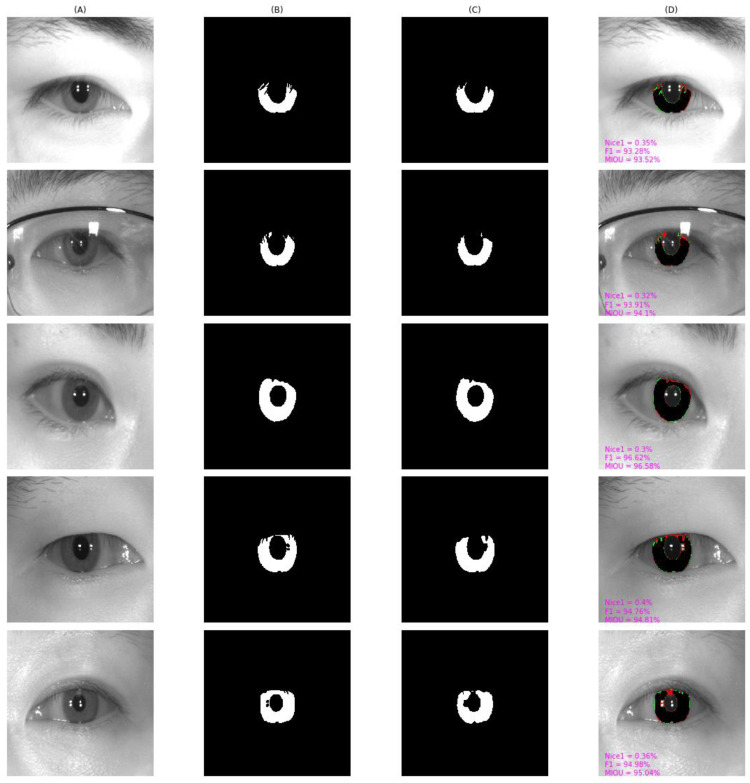
The segmentation result from CASIA-D achieved by our proposed method. (**A**) original image, (**B**) Groundtruth mask, (**C**) segmentation mask, (**D**) segmentation result, whose green pixels, red pixels, and black pixels represent false positive pixels, false negative pixels, and true positive pixels, respectively.

**Figure 14:**
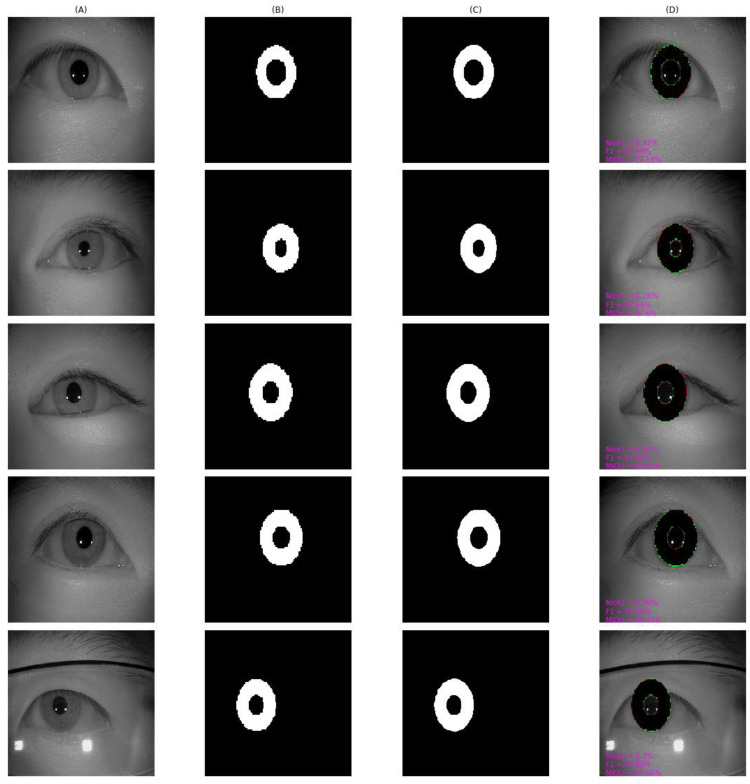
The segmentation result from CASIA-T achieved by our proposed method. (**A**) original image, (**B**) Groundtruth mask, (**C**) segmentation mask, (**D**) segmentation result, whose green pixels, red pixels, and black pixels represent false positive pixels, false negative pixels, and true positive pixels, respectively.

As shown in Figure 10, Figure 11, Figure 12, Figure 13 and Figure 14, we visualized the segmentation results of the proposed method for some samples in UBIRIS.v2, IITD, MICHE-I, CASIA-D, and CASIA-T datasets. To compare the segmentation results of the proposed algorithm with the corresponding GroundTruth masks more intuitively in these plots, the first column represents the original image, the second column represents the corresponding GroundTruth mask, the third column represents the segmentation mask, and the fourth column represents the segmentation results. In the fourth column, we marked in red the pixels that belonged to an iris in the GroundTruth mask and were incorrectly classified as a non-iris by the proposed algorithm, we marked in green the pixels that belonged to a non-iris and were incorrectly classified as an iris by the proposed algorithm, and we marked in black the pixels that belonged to an iris and were correctly classified as an iris by the proposed algorithm. The red marked pixels were called false negative pixels, the green marked pixels were called false positive pixels and the black marked pixels were called true positive pixels. Moreover, we labeled the segmentation metrics *Nice1*, *F1*, and *MIOU* for each sample.

As can be observed in Figure 10, on UBIRIS.v2, our proposed algorithm still achieved good segmentation results even in off-axis scenarios and scenarios with occlusion with eyeglass, eyelash, and eyelid interference. *F1* and *MIOU* exceeded 97% except for the *Nice1* of the second row, which was 0.44% because the iris region accounted for a larger percentage, and the *Nice1* of other samples were all less than 0.2%.

As shown in the results obtained by our proposed algorithm on IITD in Figure 11, the misidentification rate, i.e., *Nice1*, for each image exceeded 1%. There are some possible reasons. The eyelash was not correctly labeled in the manually labeled GroundTruth, while our proposed algorithm could correctly identify eyelash interference as non-iris pixels. The Groundtruth did not correctly label the iris pixels near the non-iris region, while our proposed algorithm could correctly identify the iris pixels near the non-iris region. 

Figure 12 shows the result from MICHE-I. From the fifth row, we can observe that the segmentation result of a low quality was blurred, and the off-axis sample had a larger percentage of false positive pixels, yielding an *F1* value of 92.73%. Our proposed algorithm could correctly identify such pixels as iris pixels, while the manually labeled Groundtruth could not.

We observed the results from CASIA-D shown in Figure 13. As shown in the first and second rows, *F1* and *MIOU* were slightly lower when there was more interference information with thin eyelashes. GroundTruth did not precisely label these regions, while our proposed algorithm coudl successfully identify the region as a non-iris region.

The results of our method on CASIA-T are shown in Figure 14. Since the dataset was obtained in a cooperative environment with less noise and interference information, the results our proposed algorithm obtain were highly consistent with GroundTruth, obtaining *F1* and *MIOU* of more than 97%. It is worth noting that GroundTruth did not delicately label the iris-pupil border and the iris-sclera border as circles, so our proposed algorithm produced few false positive pixels and false negative pixels.

In summary, our method overcame the interference caused by unrestricted factors in most cases and obtained promising results.

### 4.5. Comparison with Other State-of-the-Art Iris Segmentation Methods

To further verify how advanced and encouraging the proposed method was, we compared it with a large number of state-of-the-art methods, which are divided into two main categories, one being non-deep-learning traditional methods [7,11,27,32,64,65,66,67,68], and the other being CNN-based deep learning methods [15,17,19,20,34,35,69,70,71,72]. It can be observed from Table 6, Table 7, Table 8, Table 9 and Table 10 that the deep learning methods outperformed the traditional methods, and our proposed method was the optimal method among the deep learning methods. Our proposed method overcame the problem of a limited annotated iris dataset without ImageNet pretraining and data augmentation. Moreover, it reduced the distribution gap within the no-ideal dataset to improve the performance in unconstrained and uncooperative iris acquisition conditions.

As can be seen from Table 6, on the UBIRIS.v2 dataset we obtained the best results, 0.44% in the *Nice1* metric, 96.66% in the *F1*, and 96.54% in the *MIOU*. Our proposed method outperformed the best traditional method, obtaining a great improvement over TVBM [7] of 63.64% in the *Nice1*. Our proposed method significantly outperformed the best deep learning method of Miron and Pasarica [36], which obtained the results of *Nice1* = 0.53%, *F1* = 96.14%, and *MIOU* = 92.56%.

Table 7 shows the results of the different methods on the IITD dataset. Our proposed method achieved the largest *F1* value of 98.72% and *MIOU* value of 97.88%. It is noteworthy that although our proposed method obtained the second largest *Nice1* value of 1.03%, the *MIOU* and *F1* metrics, i.e., the combination of recall and precision, provide a more comprehensive evaluation of the stability and accuracy of the segmentation model. Moreover, unlike Miron and Pasarica [36], which uses data augmentation to expand the training set, we did not expand any data, so there was a slight decrease in the *Nice1* of 0.13%. Therefore, our proposed algorithm was superior to Miron and Pasarica [36].

It can be seen from Table 8 that for the dataset MICHE-I, our proposed method achieved the highest *Nice1* value of 0.66, which was equal to IrisParseNet [15]. Our *F1* value was 0.02% higher than the previous best method, DADCNet [34]. Both IrisParseNet [15] and DADCNet [34] use the same data augmentation strategy, which expands the training set by a factor of five, whereas our proposed method did not use data augmentation and still alleviated the overfitting problem, validating the superiority of our method.

Table 9 presents the results for CASIA-D. Our proposed method, without any data augmentation expansion, obtained the same *Nice1* value as the current state-of-the-art method IrisParseNet [15] on just 296 training samples of CASIA-D and exceeded IrisParseNet by 0.02% in the *F1* metric.

Table 10 presents the results achieved from CASIA-T. Our proposed method obtained the best values of *Nice1* = 0.37%, *F1* = 97.41%, and *MIOU* = 97.28%, which significantly outperformed the best deep learning method by 0.01%, 0.77%, and 3.78%, and outperformed the best traditional method by 1.03%, 9.65%, and 19.10% in *Nice1*, *F1*, *MIOU*, respectively.

### 4.6. Storage and Computational Time

Our proposed method had 37.28 M parameters and 154.61 G FLOPs relative to the shape of 513 × 513 × 3. It occupied 149.15 MB of storage space. We used an NVIDIA Tesla V100 32 G GPU to test the inference speed. Our model needed to execute approximately 0.036 s of processing per frame. The contribution of this paper aimed to provide an alternative pipeline for training iris segmentation and the initial intention was to relieve the pressure of iris pixel annotation and to compensate for the distribution differences between non-ideal data. Therefore, the storage and computational overheads need to be further optimized, which will be the focus of our future research. 

## 5. Conclusions

In this paper, our proposed method utilized supervised contrast learning to overcome the problem of limited annotated iris datasets. Furthermore, we developed an entropy-based adversarial adaptation to reduce the distribution gap within the no-ideal dataset to improve the robustness in non-ideal environments. Extensive experiments showed our three-stage approach performed experiments that outperformed state-of-the-art deep learning methods and traditional methods on both vis-light and near-infrared light datasets.

In the future, we will consider the use of self-supervision and domain adaptation to address the more difficult multi-source heterogeneous scenes. Efficient and accurate deep learning models will be designed using methods such as neural network architecture search, knowledge distillation, and model quantization.

## Figures and Tables

**Table 1:** The summary of the related work.

Methods	Category	Characteristics
Daugman, J [9]	Boundary-based	Integro-differential operator and gradient
Wildes et al. [10]	Boundary-based	Hough transform and vote
Radman et al. [26]	Boundary-based	Integro-differential operator and Hough transform
Bendale et al. [27]	Boundary-based	Improved Hough transform by one-dimensional space
Uhl et al. [28]	Boundary-based	Adaptive Hough transform
Pundlik et al. [29]	Pixel-based	Graph cut based on entropy minimization
Banerjee et al. [30]	Pixel-based	Geometry of garbcut
Radman et al. [31]	Pixel-based	HOG-SVM and cellular automata through GrowCut
Tan et al. [32]	Boundary-based and Pixel-based	Eight-neighbor connection based clustering and integrodifferential constellation
Kumar et al. [33]	Boundary-based and Pixel-based	Random walker and graph-based modeling
Liu et al. [17]	Deep learning	HCNNS and MFCNS without pre- and post-processing
Bazrafkan et al. [18]	Deep learning	Merged networks by SPDNN at layer level
Wang et al. [15]	Deep learning	Multi-task learning and parameterized inner and outer boundaries
Chen et al. [34]	Deep learning	Mask images by the DADCNET as GTs
Wang et al. [35]	Deep learning	A light network for mobile iris segmentation
Miron et al. [36]	Deep learning	A U-Net with model downscaling

**Table 2:** Class weights of different datasets.

Dataset	Non-Iris Weight	Iris Weight
UBIRIS.v2	0.53	7.37
MICHE-I	0.52	9.94
IITD	0.70	1.72
CASIA-D	0.51	14.33
CASIA-T	0.53	4.42

**Table 3:** Impact of attention-supervised contrastive learning on segmentation performance.

Dataset	Method	PA(%)	F1(%)	MIOU(%)
UBIRIS.v2	From Scratch	98.57	88.28	88.87
	ImageNet+FineTune	**(** ↑ **0.55)99.12**	(↑4.94)93.22	(↑4.46)93.24
	SCL+FineTune	(↑0.52)99.09	**(** ↑ **5.05)93.33**	**(** ↑ **4.52)93.30**
IITD	From Scratch	95.98	93.36	91.16
	ImageNet+FineTune	(↑1.55)97.53	(↑2.32)95.95	(↑3.20)94.36
	SCL+FineTune	**(** ↑ **1.90)97.88**	**(** ↑ **2.94)96.57**	**(** ↑ **4.07)95.17**
CASIA-D	From Scratch	98.90	84.19	85.83
	ImageNet+FineTune	(↑0.40)99.30	(↑6.32)90.51	(↑5.16)90.99
	SCL+FineTune	**(** ↑ **0.45)99.35**	**(** ↑ **7.27)91.46**	**(** ↑ **5.97)91.80**
MICHE-I	From Scratch	98.85	88.50	89.15
	ImageNet+FineTune	(↑0.37)99.22	(↑3.45)91.95	(↑3.01)92.16
	SCL+FineTune	**(** ↑ **0.40)99.25**	**(** ↑ **3.73)92.23**	**(** ↑ **3.26)92.41**
CASIA-T	From Scratch	99.30	95.26	95.11
	ImageNet+FineTune	(↑0.13)99.43	(↑0.86)96.13	(↑0.87)95.98
	SCL+FineTune	**(** ↑ **0.14)99.44**	**(** ↑ **0.91)96.17**	**(** ↑ **0.91)96.02**

**Table 4:** The impact of intra-dataset adaptation on segmentation performance.

Dataset	Method	PA(%)	F1(%)	MIOU(%)
UBIRIS.v2	From Scratch	98.57	88.28	88.87
	From Scratch+GLA	(↑0.28)98.85	(↑2.99)91.27	(↑2.55)91.42
	From Scratch+CLA	**(** ↑ **0.56)99.13**	**(** ↑ **4.53)92.81**	**(** ↑ **4.03)92.90**
IITD	From Scratch	95.98	93.36	91.16
	From Scratch+GLA	(↑0.97)96.95	(↑1.31)94.94	(↑1.91)93.07
	From Scratch+CLA	**(** ↑ **1.27)97.25**	**(** ↑ **1.88)95.51**	**(** ↑ **2.67)93.83**
CASIA-D	From Scratch	98.90	84.19	85.83
	From Scratch+GLA	(↑0.25)99.15	(↑3.89)88.08	(↑3.12)88.95
	From Scratch+CLA	**(** ↑ **0.29)99.19**	**(** ↑ **4.50)88.69**	**(** ↑ **3.64)89.47**
MICHE-I	From Scratch	98.85	88.50	89.15
	From Scratch+GLA	(↑0.23)99.08	(↑2.43)90.93	**(**↑2.08)91.23
	From Scratch+CLA	**(** ↑ **0.33)99.18**	**(** ↑ **3.05)91.55**	**(** ↑ **2.66)91.81**
CASIA-T	From Scratch	99.30	95.26	95.11
	From Scratch+GLA	(↑0.03)99.33	(↑0.25)95.51	(↑0.24)95.35
	From Scratch+CLA	(↑**0.06)99.36**	**(** ↑ **0.41)95.67**	**(** ↑ **0.41)95.52**

**Table 5:** Impact of different strategies on segmentation performance.

Dataset	Method			PA(%)	F1(%)	MIOU(%)
	SCL	GLA	CLA			
UBIRIS.v2	✗	✗	✗	98.57	88.28	88.87
	✓	✗	✗	(↑0.52)99.09	(↑5.05)93.33	(↑4.52)93.30
	✗	✓	✗	(↑0.28)98.85	(↑2.99)91.27	(↑2.55)91.42
	✗	✗	✓	(↑0.56)99.13	(↑4.53)92.81	(↑4.03)92.90
	✓	✓	✗	(↑0.79)99.36	(↑7.18)95.46	(↑6.45)95.32
	✓	✗	✓	**(** ↑ **0.99)99.56**	**(** ↑ **8.38)96.66**	**(** ↑ **7.67)96.54**
IITD	✗	✗	✗	95.98	93.36	91.16
	✓	✗	✗	(↑1.90)97.88	(↑2.94)96.57	(↑4.07)95.17
	✗	✓	✗	(↑0.97)96.95	(↑1.31)94.94	(↑1.91)93.07
	✗	✗	✓	(↑1.27)97.25	(↑1.88)95.51	(↑2.67)93.83
	✓	✓	✗	(↑2.30)98.28	(↑3.86)97.22	(↑4.91)96.07
	✓	✗	✓	**(** ↑ **2.99)98.97**	**(** ↑ **5.36)98.72**	**(** ↑ **6.72)97.88**
MICHE-I	✗	✗	✗	98.85	88.50	89.15
	✓	✗	✗	(↑0.40)99.25	(↑3.73)92.23	(↑3.26)92.41
	✗	✓	✗	(↑0.23)99.08	(↑2.43)90.93	(↑2.08)91.23
	✗	✗	✓	(↑0.33)99.18	(↑3.05)91.55	(↑2.66)91.81
	✓	✓	✗	(↑0.44)99.29	(↑4.26)92.76	(↑3.74)92.89
	✓	✗	✓	**(** ↑ **0.49)99.34**	**(** ↑ **4.71)93.21**	**(** ↑ **4.11)93.26**
CASIA-D	✗	✗	✗	98.90	84.19	85.83
	✓	✗	✗	(↑0.45)99.35	(↑7.27)91.46	(↑5.97)91.80

**Table 6:** The performance of different algorithms on UBIRIS.V2.

Dataset	Method	Nice1 (%)	F1(%)	MIOU(%)
UBIRIS.v2	Osiris [64]	N/A	18.65	N/A
	WAHET [28]	N/A	23.68	N/A
	IFFP [65]	N/A	28.52	N/A
	GST [66]	N/A	39.93	N/A
	TVBM [7]	1.21	N/A	N/A
	MFCN [17]	0.90	N/A	N/A
	FCDNN [16]	N/A	93.90	N/A
	DADCNet [34]	N/A	96.14	N/A
	IrisParseNet [15]	0.84	91.78	N/A
	Wang and Meng [35]	0.70	N/A	95.35
	FCEDN-Bay [70]	3.06	84.07	72.51
	Miron and Pasarica [36]	0.53	96.14	92.56
	**Ours**	**0.44**	**96.66**	**96.54**

**Table 7:** The performance of different algorithms on IITD.

Dataset	Method	Nice1(%)	F1(%)	MIOU(%)
IITD	Osiris [64]	4.37	92.23	85.52
	WAHET [28]	N/A	87.02	N/A
	IFFP [65]	N/A	85.83	N/A
	GST [66]	N/A	86.6	N/A
	DADCNet [34]	N/A	98.43	N/A
	IrisSeg [68]	N/A	94.37	N/A
	FCEDN-B [70]	5.39	84.92	80.05
	IrisDenseNet [19]	N/A	97.56	N/A
	FRED-Net [20]	N/A	97.61	N/A
	RefineNet [71]	1.50	97.40	94.93
	Miron and Pasarica [36]	**0.90**	98.48	97.09
	**Ours**	1.03	**98.72**	**97.88**

**Table 8:** The performance of different algorithms on MICHE-I.

Dataset	Method	Nice1(%)	F1(%)	MIOU(%)
MICHE-I	DADCNet [34]	N/A	93.19	N/A
	TVBM [7]	1.21	79.24	N/A
	Haindl and Krupička [67]	3.86	70.17	N/A
	MFCN [17]	0.74	92.01	N/A
	RefineNet [71]	0.80	91.41	N/A
	IrisParseNet [15]	0.66	93.05	N/A
	**Ours**	**0.66**	**93.21**	**93.26**

**Table 9:** The performance of different algorithms on CASIA-D.

Dataset	Method	Nice1(%)	F1(%)	MIOU(%)
CASIA-D	Tan and Kumar TIP2012 [11]	1.13	N/A	N/A
	RefineNet [71]	0.56	92.27	N/A
	Tan and Kumar TIP2013 [33]	0.81	N/A	N/A
	TVBM [7]	0.68	87.55	N/A
	MFCN [17]	0.59	93.09	N/A
	IrisParseNet [15]	0.41	94.25	N/A
	**Ours**	**0.41**	**94.28**	**94.38**

**Table 10:** The performance of different algorithms on CASIA-T.

Dataset	Method	Nice1(%)	F1(%)	MIOU(%)
CASIA-T	Osiris [64]	1.34	87.76	78.18
	IrisSeg [68]	0.95	91.39	84.14
	Miron and Pasarica [36]	0.38	96.64	93.50
	FCN [72]	0.61	94.42	89.42
	FCDNN [16]	N/A	95.94	N/A
	**Ours**	**0.37**	**97.41**	**97.28**

## Data Availability

The data used to support the findings of this study have been deposited in the http://iris.di.ubi.pt/ubipr.html, http://biplab.unisa.it/MICHE/index_miche.htm, https://www4.comp.polyu.edu.hk/~csajaykr/IITD/Database_Iris.htm, http://biometrics.idealtest.org/#/datasetDetail/4. In this paper, the literature [58,59,60,61] are cited in the references. Readers can download the data by clicking on the link above.

## References

[1] Li C., Zhou W., Yuan S. (2015). Iris recognition based on a novel variation of local binary pattern. Visual Comput..

[2] Ma L., Tan T., Wang Y., Zhang D. (2004). Efficient iris recognition by characterizing key local variations. IEEE Trans. Image Process..

[3] Wang C., Sun Z. (2020). A Benchmark for Iris Segmentation. J. Comput. Res. Dev..

[4] (1999). Biometrics: Personal Identification in Networked Society.

[5] Umer S., Dhara B.C., Chanda B. (2019). NIR and VW iris image recognition using ensemble of patch statistics features. Visual Comput..

[6] He Z., Tan T., Sun Z., Qiu X. (2009). Toward Accurate and Fast Iris Segmentation for Iris Biometrics. IEEE Trans. Pattern Anal. Mach. Intell..

[7] Zhao Z., Ajay K. An accurate iris segmentation framework under relaxed imaging constraints using total variation model. Proceedings of the IEEE International Conference on Computer Vision.

[8] Hofbauer H., Alonso-Fernandez F., Bigun J., Uhl A. (2016). Experimental analysis regarding the influence of iris segmentation on the recognition rate. IET Biom..

[9] Daugman J. (2007). New methods in iris recognition. IEEE Trans. Syst. Man Cybern. B Cybern.

[10] Wildes R.P. (1997). Iris Recognition: An Emerging Biometric Technology. Proc.-IEEE.

[11] Tan C.W., Kumar A. (2012). Unified framework for automated iris segmentation using distantly acquired face images. IEEE Trans. Image Proc..

[12] Proenca H. (2009). Iris recognition: On the segmentation of degraded images acquired in the visible wavelength. IEEE Trans. Pattern Anal. Mach. Intell..

[13] Deng J., Dong W., Socher R., Li L.J., Li K., Fei-Fei L. (2009). Imagenet: A large-scale hierarchical image database. Proceedings of the 2009 IEEE Conference on Computer Vision and Pattern Recognition.

[14] Raghu M., Zhang C., Kleinberg J., Bengio S. (2019). Transfusion: Understanding Transfer Learning for Medical Imaging. Adv. Neural Inf. Process. Syst..

[15] Wang C., Muhammad J., Wang Y., He Z., Sun Z. (2020). Towards complete and accurate iris segmentation using deep multi-task attention network for non-cooperative iris recognition. IEEE Trans. Inf. Forensics Secur..

[16] Jan F., Alrashed S., Min-Allah N. (2021). Iris segmentation for non-ideal Iris biometric systems. Multimed. Tools Appl..

[17] Liu N., Li H., Zhang M., Liu J., Sun Z., Tan T. (2016). Accurate iris segmentation in non-cooperative environments using fully convolutional networks. Proceedings of the 2016 International Conference on Biometrics (ICB).

[18] Bazrafkan S., Thavalengal S., Corcoran P. (2018). An end to end deep neural network for iris segmentation in unconstrained scenarios. Neural Netw..

[19] Arsalan M., Naqvi R.A., Kim D.S., Nguyen P.H., Owais M., Park K.R. (2018). IrisDenseNet: Robust iris segmentation using densely connected fully convolutional networks in the images by visible light and near-infrared light camera sensors. Sensors.

[20] Arsalan M., Kim D.S., Lee M.B., Park K.R. (2019). FRED-Net: Fully residual encoder–decoder network for accurate iris segmentation. Expert Syst. Appl..

[21] Lakra A., Tripathi P., Keshari R., Vatsa M., Singh R. (2018). Segdensenet: Iris segmentation for pre-and-post cataract surgery. Proceedings of the 2018 24th International Conference on Pattern Recognition (ICPR).

[22] He K., Fan H., Wu Y., Xie S., Girshick R. Momentum contrast for unsupervised visual representation learning. Proceedings of the IEEE/CVF Conference on Computer Vision and Pattern Recognition.

[23] Chen T., Kornblith S., Norouzi M., Hinton G. A simple framework for contrastive learning of visual representations. Proceedings of the International Conference on Machine Learning PMLR. Virtual Event.

[24] Pan F., Shin I., Rameau F., Lee S., Kweon I.S. Unsupervised intra-domain adaptation for semantic segmentation through self-supervision. Proceedings of the IEEE/CVF Conference on Computer Vision and Pattern Recognition.

[25] Vu T.H., Jain H., Bucher M., Cord M., Pérez P. Advent: Adversarial entropy minimization for domain adaptation in semantic segmentation. Proceedings of the IEEE/CVF Conference on Computer Vision and Pattern Recognition.

[26] Radman A., Jumari K., Zainal N. (2012). Iris segmentation in visible wavelength environment. Procedia Eng..

[27] Bendale A., Nigam A., Prakash S., Gupta P. (2012). Iris segmentation using improved hough transform. Emerging Intelligent Computing Technology and Applications.

[28] Uhl A., Wild P. (2012). Weighted adaptive Hough and ellipsopolar transforms for real-time iris segmentation. Proceedings of the 2012 5th IAPR international conference on biometrics (ICB).

[29] Pundlik S.J., Woodard D.L., Birchfield S.T. (2008). Non-ideal iris segmentation using graph cuts. Proceedings of the 2008 IEEE Computer Society Conference on Computer Vision and Pattern Recognition Workshops.

[30] Banerjee S., Mery D. (2015). Iris segmentation using geodesic active contours and grabcut. Image and Video Technology.

[31] Radman A., Zainal N., Suandi S.A. (2017). Automated segmentation of iris images acquired in an unconstrained environment using HOG-SVM and GrowCut. Digit. Signal Processing.

[32] Tan T., He Z., Sun Z. (2010). Efficient and robust segmentation of noisy iris images for non-cooperative iris recognition. Image Vision Comput..

[33] Tan C.W., Kumar A. (2013). Towards online iris and periocular recognition under relaxed imaging constraints. IEEE Trans. Image Process..

[34] Chen Y., Gan H., Zeng Z., Chen H. (2021). DADCNet: Dual attention densely connected network for more accurate real iris region segmentation. Int. J. Intell. Syst..

[35] Wang Q., Meng X., Sun T., Zhang X. (2021). A light iris segmentation network. Visual Comput..

[36] Miron C., Pasarica A., Manta V., Timofte R. (2022). Efficient and robust eye images iris segmentation using a lightweight U-net convolutional network. Multimed. Tools Appl..

[37] Long J., Shelhamer E., Darrell T. Fully convolutional networks for semantic segmentation. Proceedings of the IEEE Conference on Computer Vision and Pattern Recognition.

[38] Chen L.C., Papandreou G., Schroff F., Adam H. (2017). Rethinking atrous convolution for semantic image segmentation. arXiv.

[39] Sun K., Xiao B., Liu D., Wang J. Deep high-resolution representation learning for human pose estimation. Proceedings of the IEEE/CVF Conference on Computer Vision and Pattern Recognition.

[40] Yuan Y., Chen X., Wang J. Object-contextual representations for semantic segmentation. Proceedings of the Computer Vision–ECCV 2020: 16th European Conference.

[41] Zhang X., Xu H., Mo H., Tan J., Yang C., Wang L., Ren W. Dcnas: Densely connected neural architecture search for semantic image segmentation. Proceedings of the IEEE/CVF Conference on Computer Vision and Pattern Recognition.

[42] Ramana K., Kumar M.R., Sreenivasulu K., Gadekallu T.R., Bhatia S., Agarwal P., Idrees S.M. (2022). Early prediction of lung cancers using deep saliency capsule and pre-trained deep learning frameworks. Front. Oncol..

[43] Gao Y., Zhou M., Metaxas D.N. (2021). UTNet: A hybrid transformer architecture for medical image segmentation. International Conference on Medical Image Computing and Computer-Assisted Intervention.

[44] Cheng B., Misra I., Schwing A.G., Kirillov A., Girdhar R. Masked-attention mask transformer for universal image segmentation. Proceedings of the IEEE/CVF Conference on Computer Vision and Pattern Recognition.

[45] Ma J., Chen J., Ng M., Huang R., Li Y., Li C., Yang X., Martel A.L. (2021). Loss odyssey in medical image segmentation. Med. Image Anal..

[46] Liu X., Guo Z., Li S., Xing F., You J., Kuo C.C.J., El Fakhri G., Woo J. Adversarial unsupervised domain adaptation with conditional and label shift: Infer, align and iterate. Proceedings of the IEEE/CVF International Conference on Computer Vision.

[47] Yan L., Fan B., Xiang S., Pan C. (2021). CMT: Cross Mean Teacher Unsupervised Domain Adaptation for VHR Image Semantic Segmentation. IEEE Geosci. Remote Sens. Lett..

[48] Fan B., Yang Y., Feng W., Wu F., Lu J., Liu H. (2022). Seeing through Darkness: Visual Localization at Night via Weakly Supervised Learning of Domain Invariant Features. IEEE Trans. Multimed..

[49] Hu J., Shen L., Sun G. Squeeze-and-excitation networks. Proceedings of the IEEE Conference on Computer Vision and Pattern Recognition.

[50] Hadsell R., Chopra S., LeCun Y. (2006). Dimensionality reduction by learning an invariant mapping. Proceedings of the 2006 IEEE Computer Society Conference on Computer Vision and Pattern Recognition (CVPR’06).

[51] Hjelm R.D., Fedorov A., Lavoie-Marchildon S., Grewal K., Bachman P., Trischler A., Bengio Y. (2018). Learning deep representations by mutual information estimation and maximization. arXiv.

[52] Bachman P., Hjelm R.D., Buchwalter W. (2019). Learning representations by maximizing mutual information across views. arXiv.

[53] Chen X., He K. Exploring simple siamese representation learning. Proceedings of the IEEE/CVF Conference on Computer Vision and Pattern Recognition.

[54] He K., Zhang X., Ren S., Sun J. Deep residual learning for image recognition. Proceedings of the IEEE conference on Computer Vision and Pattern Recognition.

[55] Shannon C.E. (1948). A mathematical theory of communication. Bell Syst. Tech. J..

[56] Chen C., Xie W., Huang W., Rong Y., Ding X., Huang Y., Xu T., Huang J. Progressive feature alignment for unsupervised domain adaptation. Proceedings of the IEEE/CVF Conference on Computer Vision and Pattern Recognition.

[57] Wang H., Shen T., Zhang W., Duan L.Y., Mei T. (2020). Classes matter: A fine-grained adversarial approach to cross-domain semantic segmentation. Proceedings of the European Conference on Computer Vision.

[58] De Marsico M., Nappi M., Riccio D., Wechsler H. (2015). Mobile iris challenge evaluation (MICHE)-I, biometric iris dataset and protocols. Pattern Recognit. Lett..

[59] Proença H., Filipe S., Santos R., Oliveira J., Alexandre L.A. (2009). The UBIRIS. v2: A database of visible wavelength iris images captured on-the-move and at-a-distance. IEEE Trans. Pattern Anal. Mach. Intell..

[60] Kumar A., Passi A. (2010). Comparison and combination of iris matchers for reliable personal authentication. Pattern Recognit..

[61] Chinese Academy of Sciences Institute of Automation Casia Iris Image Databases. http://biometrics.idealtest.org/#/datasetDetail/4.

[62] Proença H., Alexandre L.A. (2007). The nice. i: Noisy iris challenge evaluation-part i. Proceedings of the 2007 First IEEE International Conference on Biometrics: Theory, Applications, and Systems.

[63] Badrinarayanan V., Kendall A., Cipolla R. (2017). Segnet: A deep convolutional encoder-decoder architecture for image segmentation. IEEE Trans. Pattern Anal. Mach. Intell..

[64] Petrovska D., Mayoue A. (2009). Description and Documentation of the Biosecure Software Library.

[65] Uhl A., Wild P. (2012). Multi-stage visible wavelength and near infrared iris segmentation framework. Image Analysis and Recognition.

[66] Alonso-Fernandez F., Bigun J. (2012). Iris boundaries segmentation using the generalized structure tensor. A study on the effects of image degradation. Proceedings of the 2012 IEEE Fifth International Conference on Biometrics: Theory, Applications and Systems (BTAS).

[67] Haindl M., Krupička M. (2015). Unsupervised detection of non-iris occlusions. Pattern Recognit. Lett..

[68] Gangwar A., Joshi A., Singh A., Alonso-Fernandez F., Bigun J. (2016). IrisSeg: A fast and robust iris segmentation framework for non-ideal iris images. Proceedings of the 2016 International Conference on Biometrics (ICB).

[69] Varkarakis V., Bazrafkan S., Corcoran P. (2020). Deep neural network and data augmentation methodology for off-axis iris segmentation in wearable headsets. Neural Netw..

[70] Jalilian E., Uhl A. (2017). Iris segmentation using fully convolutional encoder–decoder networks. Deep Learning for Biometrics.

[71] Hofbauer H., Jalilian E., Uhl A. (2019). Exploiting superior CNN-based iris segmentation for better recognition accuracy. Pattern Recognit. Lett..

[72] Bezerra C.S., Laroca R., Lucio D.R. (2018). Robust iris segmentation based on fully convolutional networks and generative adversarial networks. Proceedings of the 2018 31st SIBGRAPI Conference on Graphics, Patterns and Images (SIBGRAPI).

